# Boltzmann Thermometry at Cryogenic Temperatures Exploiting Stark Sublevels in Er^3+^/Yb^3+^-Codoped Yttrium Oxide Nanoparticles

**DOI:** 10.1021/acsami.5c21528

**Published:** 2026-01-19

**Authors:** Thomas Possmayer, Allison R. Pessoa, Jefferson A. O. Galindo, Luiz F. dos Santos, Rogéria R. Gonçalves, Anderson M. Amaral, Leonardo de S. Menezes

**Affiliations:** † Chair in Hybrid Nanosystems, Faculty of Physics, 9183 Ludwig-Maximilians-Universität München, 80539 München, Germany; ‡ Federal Institute of Education, Science and Technology of Pernambuco, 50740-545 Recife, PE, Brazil; § Department of Physics, 28116 Universidade Federal de Pernambuco, 50670-901 Recife, PE, Brazil; ∥ Department of Chemistry, Center of Nanotechnology and Tissue Engineering—Mater Lumen Laboratory, Faculty of Philosophy, Science and Letters of Ribeirão Preto, 28133 University of São Paulo, 14040-901 Ribeirão Preto, SP, Brazil

**Keywords:** lanthanide ions, stark sublevels, Boltzmann thermometry, cryogenic optical thermometers, optical nanothermometry

## Abstract

The development of reliable luminescent nanothermometers for cryogenic applications is essential for advancing quantum technologies, superconducting systems, and other fields that require precise, high-spatial-resolution temperature monitoring. Lanthanide-doped systems are vastly employed to this purpose, and typically perform optimally at or above room temperature when manifold-to-manifold transitions are used. In this work we exploit individual Stark sublevels to demonstrate an optical Boltzmann thermometer based on Er^3+^/Yb^3+^ codoped yttria (Y_2_O_3_) nanoparticles that operates effectively across the temperature range from 25 to 175 K. This is achieved due to the pronounced crystal field environment of the Y_2_O_3_ host matrix, leading to well-separated Stark lines in the luminescence spectrum of the Er^3+^ ions. By applying the Luminescence Intensity Ratio (LIR) method to transitions originating from two Stark components of the ^4^S_3/2_ manifold of the Er^3+^ ions, we achieve thermal sensitivities up to 1.22% K^–1^ at 100 K and temperature resolutions reaching 0.6 K. Our results further experimentally confirm recently published theoretical predictions, demonstrating that thermometric performance is not directly dependent on the peak energy separation of the resulting spectral lines of the involved electronic energy levels when using individual Stark transitions to evaluate the LIR. The proposed procedure gives an energy gap calibration that matches the one determined by sample spectroscopy for nonoverlapping lines in the luminescence spectrum. These insights provide a robust foundation for the design of high-performance cryogenic thermometers based on rare-earth-doped materials.

## Graphical abstract

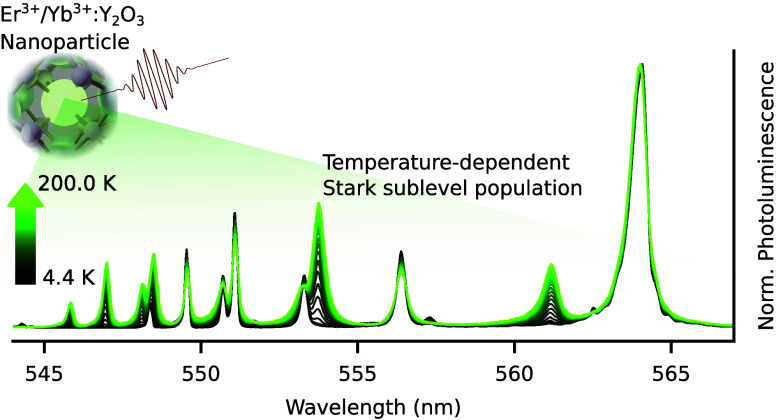

## Introduction

The emergence of quantum technologies and the growing demand for precise cryogenic temperature control (at temperature ranges below 140 K) have intensified the need for reliable nanometer- to submicrometer-sized cryothermometers. These sensors are applicable in areas that go beyond quantum computing,^1^ reaching the aerospace industry,^2^ superconductivity-based devices,^3^ and even medicine and cryobiology.^4^ Lanthanide ion (Ln^3+^)-doped nanoparticles offer a promising platform for nanoscale and noninvasive optical temperature measurements, showing potential to achieve thermal resolutions below 0.1 K.^5^

Luminescence thermometry based on such nanoparticles has been extensively studied, particularly within or close to the biological temperature range (0 to 50 °C).^6,7^ The conventional approach relies on the Luminescence Intensity Ratio (LIR) technique involving two thermally coupled (TC) spin–orbit energy manifolds of the Ln^3+^ ions. This method exploits the Boltzmann distribution governing the relative electronic population of the ions’ energy levels,^5^ which leads to a temperature-dependent emission ratio. Thermometric characteristics such as thermal sensitivity and resolution are directly connected to the energy difference between the TC manifolds.^5^ For instance, Suta and Meijerink showed that the most responsively detected temperature is *T*_opt_ = Δ*E*/(2*k*_B_), where Δ*E* is the manifold energy separation and *k*_B_ is Boltzmann’s constant.^8^ More recently, Pessoa et al. have shown theoretically that this energy separation should be treated as an effective value, Δ*E*_eff_, which also incorporates the oscillator strengths of the electronic transitions involved.^9^

For most Ln^3+^-doped materials, the effective energy difference between spin–orbit manifolds relevant for thermometry is on the order of 10^3^ cm^–1^.^10,11^ Specifically, for Er^3+^-based sensors, where the ^2^H_11/2_ and ^4^S_3/2_ manifolds form a TC pair around room temperature, this separation typically ranges from 650 cm^–1^ to 850 cm^–1^, depending on the host matrix and experimental conditions.^9^ As a result, Boltzmann-type thermometers based on these spin–orbit manifolds operate optimally in the 273 to 600 K range.^8^

To meet the growing need for cryogenic-range luminescent thermometers, several studies have proposed alternative approaches that exploit the temperature-dependent luminescent behavior of non-TC levels. These include mechanisms such as temperature-dependent nonradiative energy transfer,^12–14^ phonon-assisted energy migration,^15^ and variations in luminescence decay time.^16^ Only recently, some works have turned toward exploiting individual Stark sublevels within the TC manifolds.^17–25^ These Stark sublevels arise from the splitting of otherwise degenerate spin–orbit levels due to local electric fields in the crystal environment.^10^ With typical separations on the order of 10^2^ cm^–1^, they enable optimal thermal responses around 40 K.^8^ However, the applicability of this approach depends strongly on the host matrix, as it must induce sufficiently large and spectrally resolved Stark splittings. Yttria (Y_2_O_3_) is particularly well-suited for this purpose, as it produces pronounced Stark splitting and narrow emission lines from embedded Ln^3+^ ions.^26,27^

While optical cryothermometers based on Stark sublevels have been proposed, a comprehensive understanding of the factors governing their sensitivity and accuracy remains unavailable. A recent theoretical framework has exposed and discussed some of these problems.^9^ Specifically, it has been shown that the energy difference between the barycenters of two given Stark lines does not necessarily determine thermal characteristics of the thermometer, such as its relative sensitivity and accuracy, as they do not represent the real relative population distribution and energy difference between the TC Stark sublevels.^9^

In this work, we leverage this insight to select appropriate lines that avoid spectral overlapping and intruding bands, both of which are known to degrade the accuracy of ratiometric thermometry, as already demonstrated for manifold-to-manifold transitions.^26^ We demonstrate a Boltzmann-type optical thermometer operating in the cryogenic range between 25 and 175 K based on selected Stark-to-Stark transitions of Er^3+^ ions, from the ^4^S_3/2_ manifold to the ground state (^4^I_15/2_). Our results validate recently published theoretical predictions^9^ and provide a step forward to achieve accurate and high-precision cryogenic thermometry using rare-earth-doped systems.

## Results and Discussion

### Upconversion Spectrum at Low Temperatures

In thermometry experiments employing Yb^3+^/Er^3+^ codoped systems, it is common to excite the TC levels via a two-photon upconversion scheme: Illumination at 980 nm initially populates the ^4^F_7/2_ manifold of Er^3+^ ions through a two-step upconversion process assisted by the Yb^3+^ ions in a well-known mechanism of donor–acceptor energy transfer.^28^ Following the excitation, nonradiative relaxation via electron–phonon interactions transfers population from ^4^F_7/2_ to the metastable ^2^H_11/2_ and ^4^S_3/2_ manifolds. These two levels are considered TC at room temperature, as their energy separation is small enough to allow thermalization in a time scale of nanoseconds to hundreds of nanoseconds, depending on the host matrix,^9^ leading to a population probability distribution described by Boltzmann statistics. Both levels subsequently decay radiatively to the ground state (^4^I_15/2_), emitting photons in the green spectral range. The precise emission wavelengths are determined by the Stark sublevel structure of the excited and ground manifolds. Figure 1a depicts the unit cell of the used yttria matrix with an Er^3+^ ion substituting an Y^3+^ ion and Figure 1b shows the relevant photophysical processes under the considered excitation scheme (not showing the Yb^3+^ ions’ energy levels for simplicity), while Figure 1c features the Stark sublevel structure of the ^4^S_3/2_ and ground state manifolds.

**Figure 1:**
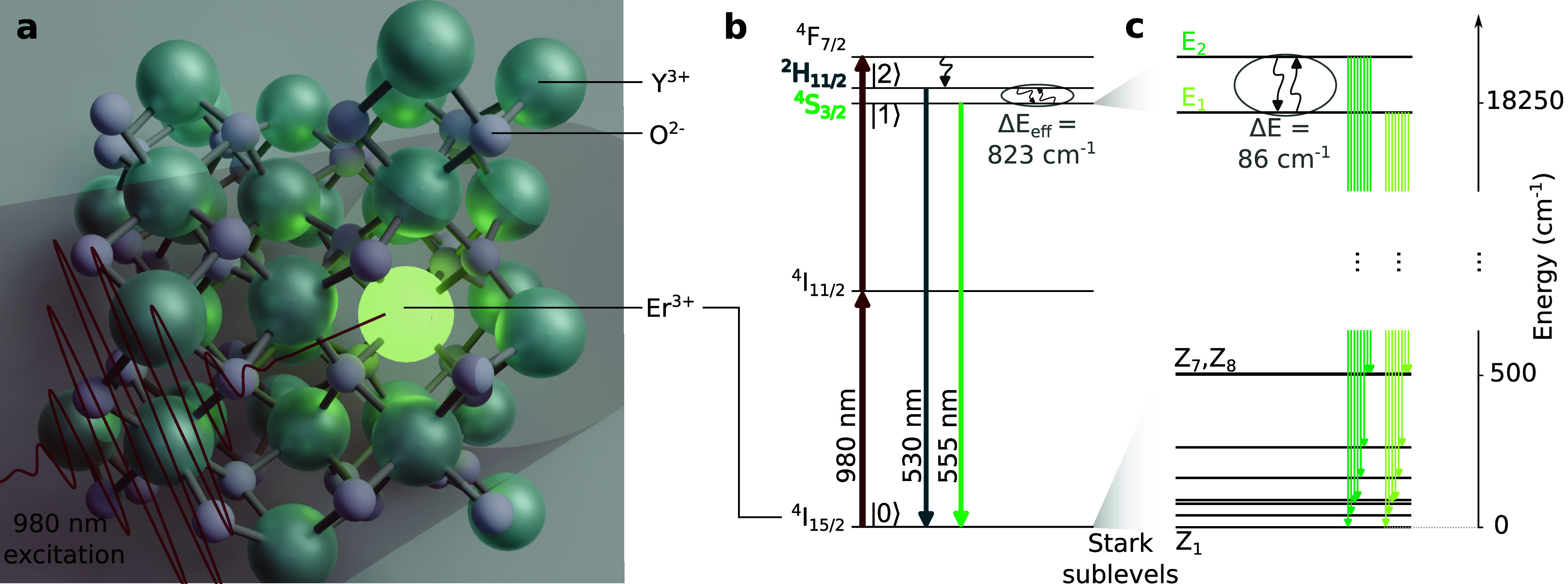
(a) Unit cell of Y_2_O_3_ with a doping Er^3+^ ion (bright green) occupying a *C*_2_ symmetry site, emitting upconverted green light under excitation at a wavelength of 980 nm. Turquoise (gray) spheres represent yttrium (oxygen) atoms. (b) Simplified energy-level diagram of the Er^3+^ ions, showing the relevant levels for nanothermometry via two-photon optical excitation. Upward straight arrows show possible energy transfer mechanisms from Yb^3+^ ions (omitted for clarity) or direct absorption from Er^3+^ ions. Downward straight arrows represent radiative decays; curly arrows indicate nonradiative relaxation processes. Circled levels are considered TC around room temperature and above. (c) Stark sublevels of the corresponding spin–orbit manifolds. The circled levels are candidates for being used as TC levels in LIR-based optical thermometry experiments performed at cryogenic temperatures.

The energy separation and remaining degeneracies of the resulting Stark sublevels are mainly governed by the host matrix. In the Y_2_O_3_ crystalline material, Er^3+^ and Yb^3+^ ions occupy sites with *C*_2_ and *C*_3*i*_ point symmetry, with the optical emission in the green spectral region predominantly stemming from the former site.^29^ Under this local symmetry, the ^2^H_11/2_ and ^4^S_3/2_ manifolds split into six and two Stark sublevels, respectively.^11^ The latter thus provides a potential further pair of TC levels at low temperatures (Figure 1c). Similarly, the ground state ^4^I_15/2_ splits into eight sublevels. Each of these Stark sublevels is a Kramer’s doublet and therefore doubly degenerate.^11^

Assuming that the electronic population in the TC manifolds follows Boltzmann statistics, the occupation probability of a Stark sublevel |*i*Γ_*k*_⟩ within the manifold |*i*⟩ is given by^9^

1 $$p_{\mathrm{ik}}\left(T\right)=\frac{g_{\mathrm{ik}}\exp\left(-\frac{E_{\mathrm{ik}}-E_{0}}{k_{B}T}\right)}{\sum_{j}\sum_{l=1}^{L_{j}}g_{\mathrm{jl}}\exp\left(-\frac{E_{\mathrm{jl}}-E_{0}}{k_{B}T}\right)}$$

where *g*_*jl*_ is the degeneracy of the Stark sublevel |*j*Γ_*l*_⟩ and *E*_*jl*_ is its energy. The reference energy *E*_0_ corresponds to the lowest Stark sublevel of the ^4^S_3/2_ manifold. *k*_B_ is Boltzmann’s constant and *T* is the absolute temperature. The sum over *j* includes both TC manifolds, while the sum over *l* runs over all *L*_*j*_ Stark sublevels within each manifold.

Temperature variation from cryogenic to room temperature therefore induces significant changes in the electronic population, and consequently also in the emission spectrum of the investigated nanoparticles (see Methods section), as shown in Figure 2a. Figure 2b shows the measured luminescence spectra at 4.4 and 305.0 K, highlighting the higher population probability of the lower-energy manifold at low temperatures. The observed spectral lines have average width in the order of 0.8 nm. Emission lines with wavelengths between 517 and 542 nm correspond to transitions from Stark sublevels of ^2^H_11/2_ to Stark sublevels of the ground state ^4^I_15/2_ (respecting possible selection rules), while those between 545 and 570 nm correspond to transition between the Stark sublevels of ^4^S_3/2_ → ^4^I_15/2_.

**Figure 2:**
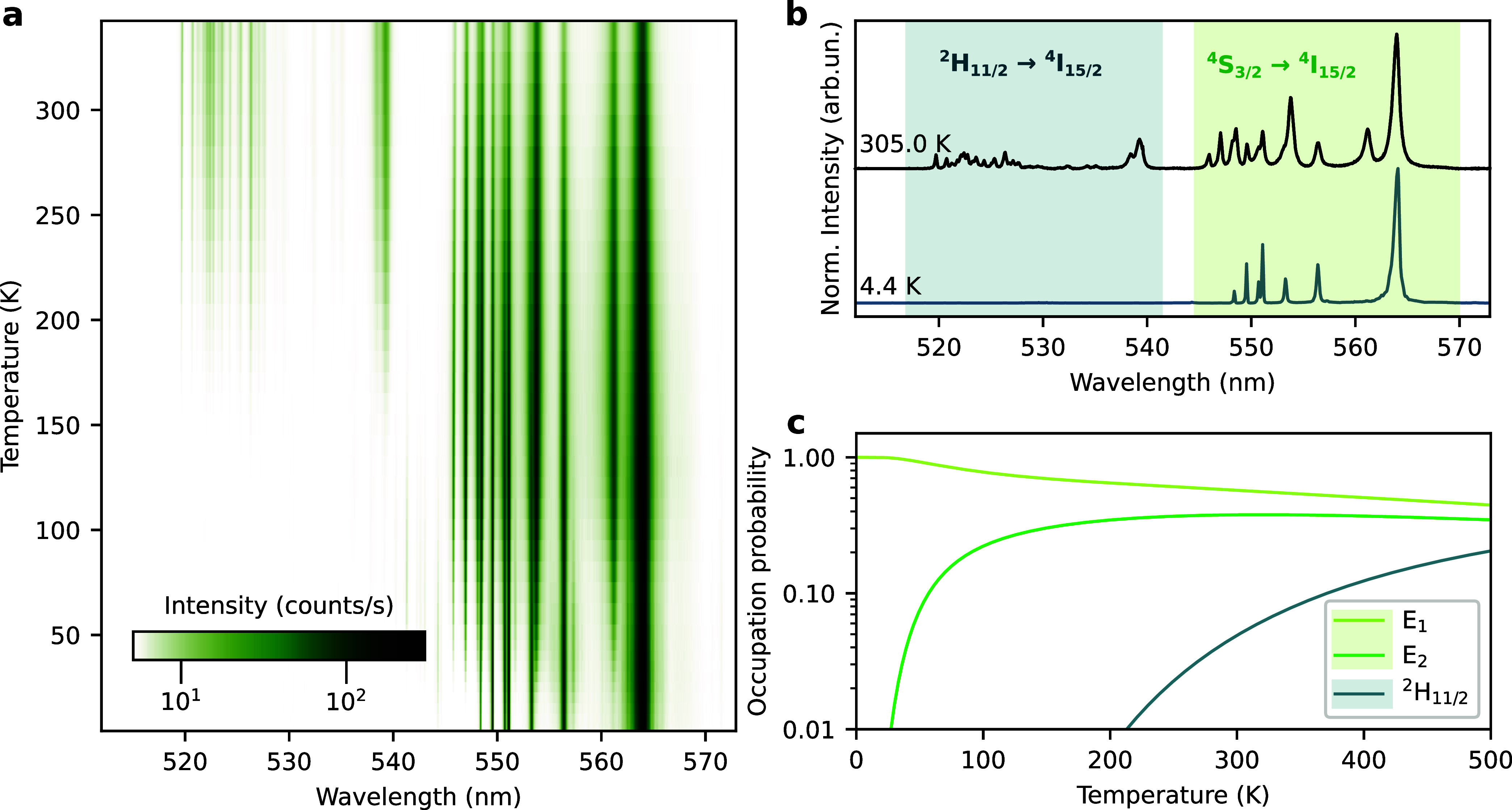
(a) Temperature-dependent spectra of the upconverted luminescence in the green spectral region emitted by Y_2_O_3_: Yb^3+^/Er^3+^ nanoparticles under 980 nm excitation. Emission from the ^2^H_11/2_ manifold is negligible at cryogenic temperatures. (b) Normalized luminescence spectra at 4.4 and 305.0 K (taken from panel a) with highlighted manifold transitions. (c) Calculated occupation probability distribution of the thermally coupled levels of Er^3+^. Labels E_1_ and E_2_ correspond to the two Stark components of the ^4^S_3/2_ manifold; ^2^H_11/2_ denotes the combined population of all Stark sublevels of the ^2^H_11/2_ manifold.

To further illustrate the temperature-dependent population in the sublevels, Figure 2c shows calculated occupation probabilities (eq 1) for the two Stark sublevels of the ^4^S_3/2_ manifold, labeled as E_1_ and E_2_ in Figure 1b (following the empirical notation), as well as the combined occupation probability of all sublevels in the ^2^H_11/2_ manifold. These calculations were performed using the levels’ energies provided by Kisliuk et al.^29^ for Er^3+^ in a Y_2_O_3_ matrix. At room temperature, the ^2^H_11/2_ manifold starts becoming appreciably populated, while E_1_ and E_2_ are close to being equally populated due to their small energy separation. At cryogenic temperatures, however, occupation of the ^2^H_11/2_ manifold is negligible, and the higher-lying E_2_ sublevel is depopulated in favor of E_1_.

### Identifying Stark Sublevels

To investigate the population distribution of the Stark sublevels by means of the Boltzmann law, we spectroscopically measured the energy separation between the Stark peaks in the luminescence spectrum. This requires transforming the spectral curve (*I*(λ)) to the energy domain, using^8^

2 $$Ĩ\left(E\right)=\left(\frac{\lambda^{2}}{\mathrm{hc}}\right)I\left(\lambda \right)$$

where *h* is Planck’s constant and *c* the speed of light in vacuum. Figure 3 shows the ^4^S_3/2_ → ^4^I_15/2_ luminescence band converted to the energy scale, along with the assignments of selected Stark–Stark transitions for two different temperatures (a broader temperature sweep is presented in Supporting Section S1). A similar analysis was performed for the ^2^H_11/2_ → ^4^I_15/2_ luminescence band at temperatures above 200 K. The extracted energies of the Stark sublevels–obtained at 140 K for ^4^S_3/2_ and at 260 K for ^2^H_11/2_–are listed in Table 1.

**Figure 3:**
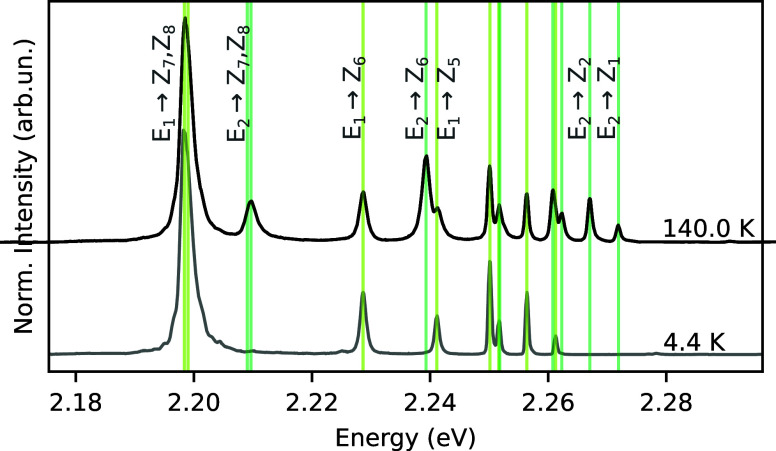
Normalized spectra of the ^4^S_3/2_ → ^4^I_15/2_ luminescence band, plotted in energy scale, at 4.4 and 140.0 K (offset for clarity). Selected transitions relevant to the thermometric analysis are labeled using empirical notation.

**Table 1:** Measured Energies of the Stark Sublevels within the Relevant Er^3+^ Spin-Orbit Manifolds in Y_2_O_3_ at 140 and 260 K for the ^4^S_3/2_ and ^2^H_11/2_ Manifolds, Respectively^a^

spin–orbit manifold	empirical notation	energy (cm^–1^)	energy (cm^–1^)
		(this work)	(ref [^29^])
^4^I_15/2_	Z_1_	0	0
	Z_2_	39	39
	Z_3_	77	76
	Z_4_	90	89
	Z_5_	162	158
	Z_6_	263	258
	Z_7_	502	500
	Z_8_	507	500
^4^S_3/2_	E_1_	18,238	18,231
	E_2_	18,324	18,318
^2^H_11/2_	F_1_	19,041	19,038
	F_2_	19,050	19,045
	F_3_	19,077	19,072
	F_4_	19,192	19,187
	F_5_	19,223	19,218
	F_6_	19,247	19,243

a Values are compared to literature data from Kisliuk et al.^29^

The energy splitting between the two Stark components of the ^4^S_3/2_ manifold was experimentally determined to be 86 cm^–1^ (see Table 1). Therefore, at 4.4 K (up to approximately ∼20.0 K), nearly all excited ions remain in the lowest Stark sublevel of ^4^S_3/2_ (E_1_), as predicted in Figure 2c. Accordingly, the luminescence spectrum at this temperature in Figure 3 exhibits only eight distinct peaks–two of which are spectrally overlapped–corresponding to radiative decays from E_1_ to the eight Stark components of the ground state ^4^I_15/2_ (Z_1_ to Z_8_). As the temperature increases and E_2_ becomes thermally populated, eight additional spectral lines emerge in the 540 nm–570 nm range. As shown in Figure 2a, above 200 K the ^2^H_11/2_ → ^4^I_15/2_ emission band also appears, while the intensity of the ^4^S_3/2_ → ^4^I_15/2_ transition begins to decrease.

Interestingly, we observed that the overall emission intensity of the ^4^S_3/2_ → ^4^I_15/2_ transition reaches a maximum near 140 K (see Section S2 of the Supporting Information). This behavior is likely attributable to mechanical shifts in the optical illumination or collection paths with varying temperature. Additionally, the number of excited ions depends on the efficiency of the upconversion process, which may itself be temperature dependent due to the thermal activation of energy transfer processes.^30^ However, as ratiometric thermometry involves comparing emission intensities at the same temperature, such variations do not compromise the accuracy or resolution of the thermometer.

### Boltzmann Thermometry Using Stark Sublevels

For the relative populations of two TC levels to be correctly described by a Boltzmann distribution, it is required that the radiative decay rates of the TC levels are much smaller than the nonradiative, phonon-assisted thermalization rate. This condition is satisfied in commonly used host matrices, since the electron–phonon interaction rate leading to thermalization is typically five orders of magnitude greater than the radiative decay rate.^31^ However, photophysical processes such as cross-relaxation, excited-state absorption, or surface quenching–if occurring at rates comparable to those of phonon-mediated relaxation–can disrupt the Boltzmann equilibrium.^32–35^ The former two are known to become relevant only at high doping concentrations,^36,37^ with excited state absorption further becoming observable at high excitation intensities. Surface quenching, on the other hand, mostly plays a role for particles smaller than 20 nm.^38,39^ By using moderate doping concentrations (see Methods section), relatively large particles around 80 nm in diameter and excitation intensities far below the saturation threshold, these disruptions can be avoided in the following characterization.

The ratiometric Boltzmann method relies on measuring the ratio of integrated intensities of two emission bands originating from thermally coupled manifolds. In standard implementations, the LIR is calculated by integrating the entire manifold-to-manifold emission bands. However, if the host matrix allows for spectral resolution of individual Stark transitions–as is the case in Y_2_O_3_: Yb^3+^/Er^3+^–then specific Stark–Stark lines can be spectrally isolated and used to compute the LIR and consequently to determine the system’s temperature.

The intensity of a spectral line is proportional to the total photon emission rate and can be obtained by integrating the luminescence signal (*I*(λ)) over the relevant wavelength range.^8^ Care must be taken to avoid the contribution of accidentally superimposed luminescence bands which are not related to the relevant TC levels, contributing to the calculation of the LIR and leading to inaccuracies in the temperature readout.^26^ If such intruding bands are identified, they can be separated a posteriori through the use of nonarbitrary methods^40^ or alternative excitation strategies.^41^

For a transition from a Stark sublevel |*i*Γ_*k*_⟩ (with *i* ∈ {^4^S_3/2_, ^2^H_11/2_} in our case) to a ground-state sublevel |^4^I_15/2_Γ_*m*_⟩ ≡ |0Γ_*m*_⟩, the integrated intensity over a wavelength interval [λ_1_, λ_2_] without overlapping spectral lines is proportional to the excited energy level’s population *n*_*ik*_ via^9^

3 $$\int_{\lambda_{1}}^{\lambda_{2}}I\left(\lambda \right)d\lambda =\eta \left({\mathrm{\lambda ̃}}_{12}\right)\Phi_{\mathrm{ik},0m}=\eta \left({\mathrm{\lambda ̃}}_{12}\right)A_{\mathrm{ik},0m}n_{\mathrm{ik}}$$

where η­(λ̃_12_) is the average detection efficiency across the chosen integration interval, incorporating system optics and the detector sensitivity. Φ_*ik*,0*m*_ is the photon emission rate in the transition. It is equal to the population in the excited Stark sublevel |*i*Γ_*k*_⟩, *n*_*ik*_, multiplied by the Einstein coefficient for the radiative transition, *A*_*ik*,0*m*_. Similarly, one can obtain the integrated intensity of a complete manifold-to-manifold transition by summing all Stark–Stark contributions.

Pessoa et al.^9^ have shown that when employing the LIR method by integrating the complete manifold-to-manifold transitions, the LIR as a function of temperature (*R*(*T*)) is a weighted sum of exponentials. This can be approximated by a single exponential with effective parameters

4 $$R{\left(T\right)}_{\mathrm{manifold}}=\frac{\sum_{\mathrm{km}}A_{\mathrm{Hk},0m}g_{\mathrm{Hk}}\exp\left(-E_{\mathrm{Hk}}/k_{B}T\right)}{\sum_{\mathrm{lm}}A_{\mathrm{Sl},0m}g_{\mathrm{Sl}}\exp\left(-E_{\mathrm{Sl}}/k_{B}T\right)}\approx C_{\mathrm{eff}}\cdot \exp\left(-\frac{\Delta E_{\mathrm{eff}}}{k_{B}T}\right)$$

where Hk and Sl label Stark sublevels of the ^2^H_11/2_ and ^4^S_3/2_ manifolds, respectively. The parameters *C*_eff_ and Δ*E*_eff_ can be predicted by expanding eq 4 around a central β_c_ = (*k*_B_ *T*_c_)^−1^ (where *T*_c_ is the central temperature of the range under consideration) and truncating to first order

5 $$\begin{array}{l} \ln C_{\mathrm{eff}}=\ln R\left(T_{c}\right)+T_{c}\cdot S_{r}\left(T_{c}\right)\\ \Delta E_{\mathrm{eff}}=S_{r}\left(T_{c}\right)\cdot k_{B}T_{c}^{2} \end{array}$$

where $S_{r}\left(T\right)=\frac{1}{R}\frac{\partial R}{\partial T}$ is the thermometer’s relative sensitivity, a common figure of merit for comparing thermometer performances.

In practical applications, *C*_eff_ and Δ*E*_eff_ are typically determined through prior calibration, by acquiring a set of emission spectra at externally measured temperatures and fitting *R*(*T*) using eq 4. However, as shown in Figure 2, the ^2^H_11/2_ manifold is scarcely populated below 200 K (less than 1% of the total population), rendering manifold-to-manifold thermometry ineffective in the cryogenic regime due to vanishing sensitivities. In contrast, Stark sublevels with energy separations on the order of 100 cm^–1^ can enable Boltzmann thermometry at these lower temperatures. In this case, the LIR between the Stark-to-Stark transitions |*i*Γ_*k*_⟩→|0Γ_*m*_⟩ and |*j*Γ_*l*_⟩→|0Γ_*m*'_⟩ is given by a single exponential function, through^9^

6 $$R{\left(T\right)}_{\mathrm{Stark}}=\frac{A_{\mathrm{ik},0m}}{A_{\mathrm{jl},0m\prime }}\frac{g_{\mathrm{ik}}}{g_{\mathrm{jl}}}\cdot \exp\left(-\frac{\Delta E_{\mathrm{ik},\mathrm{jl}}}{k_{B}T}\right)$$

where it is possible to have *i* = *j* and *k* ≠ *l*, which corresponds to using two distinct Stark sublevels from the same manifold. Here, Δ*E*_*ik*,*jl*_ is simply the actual energy separation between the TC Stark sublevels, assuming perfect Boltzmann thermalization and accurate temperature readout.

This approach allows direct extraction of microscopic quantities from *R*(*T*)_Stark_ through curve fitting. This is not possible for the manifold-to-manifold approach since the expression for Δ*E*_eff_ (eq 5) involves all oscillator strengths between the Stark sublevels of the TC manifolds and the ground state. These Stark–Stark oscillator strengths are not straightforward to calculate since 4f–4f transitions are parity-forbidden, making the use of Judd-Ofelt theory necessary, which requires other specific details about the Ln^3+^-host system.^42^ Therefore, knowing Δ*E*_eff_ does not yield direct information about microscopic parameters.

### Thermometric Characterization

According to the results presented in Table 1, the Stark sublevels E_1_ and E_2_ of the ^4^S_3/2_ manifold are separated by 86 cm^–1^, while their radiative decay to the ground state can result in spectral lines separated by more than 590 cm^–1^ due to splitting in the ground-state manifold. To calculate the bands’ intensities, we separated the Stark lines by fitting them with Voigt profiles, as shown in Supporting Section S3. Despite their differences in spectral separation, fitting the resulting *R*_Stark_(*T*) with a Boltzmann factor from eq 6 consistently yields Δ*E*_eff_ = Δ*E*_E_1_,E_2__ ≈ 86 cm^–1^, as spectroscopically determined.

This is illustrated in Figure 4a, using two LIR pairs: (i) E_2_ → (Z_1_ + Z_2_) vs E_1_ → (Z_7_ + Z_8_), which has a spectral separation of 560.6 cm^–1^; and (ii) E_2_ → (Z_1_ + Z_2_) vs E_1_ → Z_6_, which are separated by 321.3 cm^–1^ (see Figure 3 for the line assignments). A linear relationship between ln­(*R*) and 1/*T* can be seen, as expected in a population governed by Boltzmann statistics. Its slope yields the effective energy separation, which is statistically equal to 86 cm^–1^ in both cases. As this matches the intrinsic (spectroscopically measured) energy separation of the thermally coupled levels, and the linear relationship does not show significant deviations, we conclude that the Boltzmann distribution is not influenced by disrupting pathways in these pairs across the considered temperature range. Similarly, laser-induced heating plays only a minor role for the used excitation intensity of 8 kW/cm^2^. We check this by observing the manifold transitions below the saturation regime and evaluating *R*_manifold_ as a function of the excitation dynamics (see Supporting Section S4).

**Figure 4:**
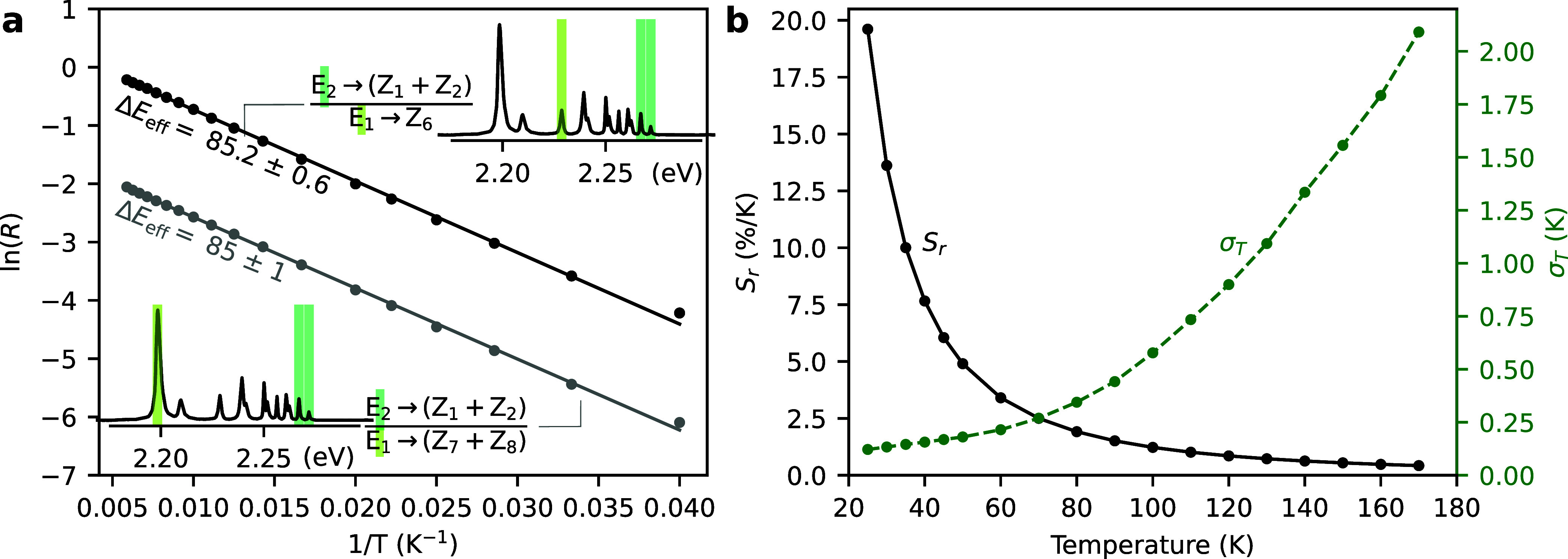
(a) Thermometric characterization using different spectral lines arising from the same thermally coupled Stark sublevels. Insets show spectra from Figure 3 at 140 K, with the transitions used for the LIR evaluation highlighted. (b) Temperature dependence of the relative sensitivity *S*_r_ and thermal resolution σ_T_ for the LIR technique using the transition E_2_ → (Z_1_ + Z_2_) and E_1_ → (Z_6_).

Here, it is worth stressing that the relative sensitivity for the Stark–Stark transitions *S*_r_ = Δ*E*_E_1_,E_2__/(*k*_B_ *T* ^2^) depends only on the true, spectroscopically measured, energy difference between the thermally coupled crystal-field states. This allows selecting the most convenient, separable lines for performing thermometry.

Another figure of merit characterizing a thermometer is the thermal resolution σ_T_, defined as the uncertainty in the temperature measurement. It is calculated via propagation in uncertainties in Δ*E*_eff_, *C*_eff_ and the measured LIR^9^

7 $$\sigma_{T}^{2}=\frac{1}{S_{r}{\left(T_{c}\right)}^{2}}\left[{\left(\frac{\sigma_{\alpha }}{T_{c}}\right)}^{2}+\sigma_{\beta }^{2}+{\left(\frac{\sigma_{R}}{R}\right)}^{2}-2\frac{\sigma_{\alpha \beta }}{T_{c}}\right]$$

where α = Δ*E*_eff_/*k*_B_, β = ln­(*C*_eff_), and σ_α_, σ_β_ and σ_R_ are the uncertainties of their corresponding variables, calculated according to Supporting Section S5. Note that σ_α_ and σ_β_ also depends on the uncertainty of the external thermometer used for calibrating the optical thermometer. σ_αβ_ is the covariance between these two variables, and it was previously shown that it can be a relevant correction.^34,35^ The resulting σ_T_ is often referred to as the precision of the temperature readout. A determination of the accuracy of the temperature would require multiple measurement methods, as only referencing it with the internal thermometer of the cryostat might introduce minor systematic errors. Therefore, it cannot be estimated in this work.^43^

Figure 4b shows the relative sensitivity and thermal resolution for a Boltzmann thermometer using the ratio between E_2_ → (Z_1_ + Z_2_) and E_1_ → (Z_6_). Table 2 presents the full thermometric characterization for selected Stark transitions and compares them to the manifold-based case.

**Table 2:** Thermometric Characterization Using Various Spectral Lines Arising from the Same Thermally Coupled Stark Sublevels, Including Their Standard Deviations (in Parentheses)^a^

LIR assignment	temperature range (K)	*C*_eff_	Δ*E*_eff_ (cm^–1^)	Δ*E*_bary_ ^b^ (cm^–1^)	Δ*E*_avg_ ^b^ (cm^–1^)	*S*_r_(*T*_c_)^b^ (% K^–1^)	σ_T_ ^b^ (K)
$\frac{E_{2}\to \left(Z_{1}+Z_{2}\right)}{E_{1}\to Z_{6}}$	25–175	1.64(2)	85.2(6)	321.3(1)	86	1.22(1)	0.6
$\frac{E_{2}\to \left(Z_{1}+Z_{2}\right)}{E_{1}\to \left(Z_{7}+Z_{8}\right)}$	25–175	0.260(4)	85(1)	560.7(1)	86	1.22(2)	1.3
$\frac{E_{2}\to \left(Z_{7}+Z_{8}\right)}{E_{1}\to \left(Z_{7}+Z_{8}\right)}$	25–175	0.37(2)	79(3)	86.8(1)	86	1.14(5)	1.6
$\frac{E_{2}\to Z_{6}}{E_{1}\to Z_{5}}$	25–175	6.7(2)	69(1)	14.9(1)	86	1.00(2)	1.0
manifold							
H11/22→I15/24S3/24→I15/24	200–350	12.4(3)	823(6)	929(1)	859	1.57(1)	1.0

a For completeness, the final row presents the corresponding results using complete manifold-to-manifold integration.

b At the central temperature of the range, *T*_c_.

These results confirm that Stark-level thermometry is viable in the 25 to 175 K range in Y_2_O_3_: Yb^3+^/Er^3+^ systems. For instance, the pair E_2_ → (Z_1_ + Z_2_) and E_1_ → Z_6_ yields high relative sensitivity in the cryogenic regime (comparable to that of using complete manifolds at room temperature), and its extracted energy separation from fitting the LIR statistically matches the true energy separation of the sublevels. Those lines are well-separated from other lines, thereby reducing artifacts in the temperature measurement related to luminescence band overlapping. In contrast, using E_2_ → (Z_1_ + Z_2_) and E_1_ → (Z_7_ + Z_8_) introduces a partial overlap with E_2_ → (Z_7_ + Z_8_), which increases uncertainties at similar sensitivities. Additionally, its lower LIR further increases the thermal uncertainty, consistent with eq 7.

The other two investigated pairs (E_2_ → (Z_7_ + Z_8_) vs E_1_ → (Z_7_ + Z_8_) and E_2_ → Z_6_ vs E_1_ → Z_5_) result in similarly high sensitivities, but feature spectrally superposed lines, which can compromise the accuracy of the extracted values; in particular, the latter pair has a peak energy separation of just 15 cm^–1^, and results in a Δ*E*_eff_ of 69 cm^–1^. This difference between the measured Δ*E*_eff_ obtained from spectrally overlapping emission lines and the true energy separation highlights the importance of choosing a host matrix that presents pronounced Stark splitting with narrow emission lines, as measured in Ln^3+^-doped yttria.

Regarding the manifold-to-manifold characterization, also shown in Table 2, we observe that Δ*E*_eff_ ≠ Δ*E*_bary_ ≠ Δ*E*_avg_, as theoretically predicted.^9^ Notably, the difference between Δ*E*_eff_ and Δ*E*_bary_ exceeds 100 cm^–1^, implying that the use of Δ*E*_bary_ instead of Δ*E*_eff_ in LIR thermometry without careful calibration may lead to temperature readout discrepancies of more than 35 K at room temperature (295 K). This was estimated by eq 7, where we have considered σ_α_ = 100/*k*_B_ and *S*_r_ = 1.36. The other uncertainties were set to zero to analyze only the influence of σ_α_ in this estimation.

## Conclusions

We have demonstrated that an optical nanothermometer based Boltzmann distribution using Y_2_O_3_: Yb^3+^/Er^3+^ nanoparticles can operate effectively in the 25 to 175 K temperature range. The luminescence spectrum of these systems exhibits well-resolved Stark lines with full widths at half-maximum around 0.8 nm and minimal spectral overlap. This spectral resolution enables the isolation of individual Stark transitions via Voigt profile fitting. By applying the LIR-based method using the two Stark sublevels of the ^4^S_3/2_ spin–orbit manifold of the Er^3+^ ions, we achieved a thermal sensitivity of 1.22% K^–1^, and a thermal resolution of 0.6 K. The energy difference between these Stark sublevels is 86 cm^–1^, measured directly through the luminescence spectra. Although their radiative decays to the Stark-split ground state result in emission lines separated by more than 590 cm^–1^, we have shown that the thermometer’s performance depends solely on the true energy separation of the TC Stark levels, independent of the resulting energy barycenter of the chosen emission lines in the luminescence spectrum. These findings confirm recent theoretical predictions concerning the principles of temperature readout in such systems, and the methodology can be extended to different host matrices and dopants that provide spectrally separable Stark lines.^9^

## Methods

### Sample Synthesis

Nanocrystalline Er^3+^/Yb^3+^ codoped Y_2_O_3_ was synthesized through a homogeneous precipitation method followed by controlled thermal treatment to ensure phase purity and crystallinity. Initially, Er^3+^ and Yb^3+^ codoped yttrium hydroxycarbonate [Y­(OH)­CO_3_·*n*H_2_O] was prepared and employed as a precursor. The homogeneous precipitation was achieved through urea thermolysis, conducted in an aqueous solution of yttrium nitrate hexahydrate (Y­(NO_3_)_3_·6H_2_O, 99.8% purity, Sigma-Aldrich) and urea (99.0% purity, Synth), with final concentrations of 0.01 mol L^–1^ and 5 mol L^–1^, respectively.

The Er^3+^ and Yb^3+^ dopants were introduced via aqueous solutions of erbium and ytterbium nitrates, which were obtained by dissolving the respective rare-earth oxides (RE_2_O_3_, RE = Er, Yb) in a slight excess of nitric acid. The acid excess was evaporated until the solution reached a pH of 4, after which the volume was adjusted to achieve a final concentration of 0.1 mol L^–1^. The dopant concentrations of Er^3+^ and Yb^3+^ were fixed at 0.5 mol % and 1.5 mol %, respectively, relative to the molar concentration of Y^3+^.

The thermolysis reaction was conducted in a sealed vessel at 80 °C for 2 h, allowing for the precipitation of the codoped precursor nanoparticles. The resulting precipitate was separated by centrifugation at 4000 rpm, washed five times with distilled water, and subsequently dried at 70 °C for 6 h. The final Er^3+^, Yb^3+^ codoped Y_2_O_3_ nanoparticles were obtained by annealing the Y­(OH)­CO_3_·*n*H_2_O precursor in air at 900 °C for 2 h, using a controlled heating rate of 5 °C min^–1^. The resulting nanoparticles had an average diameter of 80 ± 10 nm. X-ray diffraction (XRD) and Transmission Electron Microscopy (TEM) data, along with a discussion on the impact of morphology and composition on the thermometric performance, are shown in Supporting Section S6.

### Experimental Setup

The dry nanoparticle powder was compacted into a copper sample holder and placed inside a closed-cycle cryostat (Cryostation s50—Montana Instruments), capable of controlling the sample temperature between 4.4 and 350.0 K. The cryostat reference temperature has an accuracy of 5 mK at 4.4 K and 65 mK at 350 K, according to the manufacturer.

Excitation was performed using a femtosecond laser source (Chameleon Ultra II—Coherent) operating at 980 nm with an 80 MHz repetition rate and a spectral width of approximately 10 nm. A 35 mm focal length lens was placed inside the cryostat for both excitation and collection of the luminescence signal in reflection geometry (see Supporting Section S7). The excitation beam had a Gaussian profile, with an average power of 250 μW and an estimated focal area of 3.2 × 10^–8^ cm^2^, leading to an approximated excitation irradiance of 8 kW cm^–2^.

A beam splitter was used to separate the emission from the excitation light. The collected luminescence was directed into a spectrometer (Acton SP2300—Princeton Instruments), coupled to a CCD camera (Pixis 100F—Princeton Instruments). A 1800 grooves/mm diffraction grating enabled spectral resolution of individual Stark lines. The integration time for all spectra was 60 s. All temperature dependencies were measured twice with matching results, once starting from 4.4 K and ending at 350.0 K, and another one starting at 350.0 K and ending at 4.4 K, to ensure that the experimental parameters allow proper thermalization between two subsequent data points.

## Supplementary Material



## Notes

Details on sample’s characterization, experimental setup, error analysis and Stark sublevels deconvolution (PDF)
