# Supplementary material for: Boltzmann Thermometry at Cryogenic Temperatures Exploiting Stark Sublevels in Er3+/Yb3+-Codoped Yttrium Oxide Nanoparticles
Source: ACS Appl Mater Interfaces. 2026 Jan 19;18(4):7249–57. doi: 10.1021/acsami.5c21528 (PMC12884474; doi:10.1021/acsami.5c21528)
Supplement: Supplementary file 1 [file am5c21528_si_001.pdf]

Supporting Information: Boltzmann Thermometry at Cryogenic Temperatures Exploiting Stark Sublevels in Er^{3+}/Yb^{3+}-Codoped Yttrium Oxide Nanoparticles

Thomas Possmayer,† Allison R. Pessoa,*,‡,¶ Jefferson A. O. Galindo,¶ Luiz F. dos Santos,§ Rogéria R. Gonçalves,§ Anderson M. Amaral,¶ and Leonardo de S. Menezes†,¶

†*Chair in Hybrid Nanosystems, Faculty of Physics, Ludwig-Maximilians-Universität München, 80539 München, Germany*

‡*Federal Institute of Education, Science and Technology of Pernambuco, 50740-545 Recife-PE, Brazil*

¶*Department of Physics, Universidade Federal de Pernambuco, 50670-901 Recife-PE, Brazil*

§*Department of Chemistry, Center of Nanotechnology and Tissue Engineering- Mater Lumen Laboratory, Faculty of Philosophy, Science and Letters of Ribeirão Preto, University of São Paulo, 14040-901 Ribeirão Preto-SP, Brazil.*

E-mail: allison.pessoa@ufpe.br

Contents

S1 Stark-resolved spectra **S-2**

S2 Integrated intensity **S-3**

S3 Stark lines deconvolution process **S-3**

S4 Power law **S-5**

S5 Error analysis **S-5**

S6 Sample morphology and composition **S-6**

S7 Experimental setup **S-7**

S1 Stark-resolved spectra

The $^4S_{3/2} \rightarrow {}^4I_{15/2}$ luminescence band shows multiple lines, whose relative individual contributions to the overall emission depend on the temperature due to the different population of the two sublevels E_1 and E_2. By normalizing the spectra to the brightest peak ($E_1 \rightarrow (Z_7 + Z_8)$), one can observe that all E_2-originating peaks increase with temperature (Fig. S1).

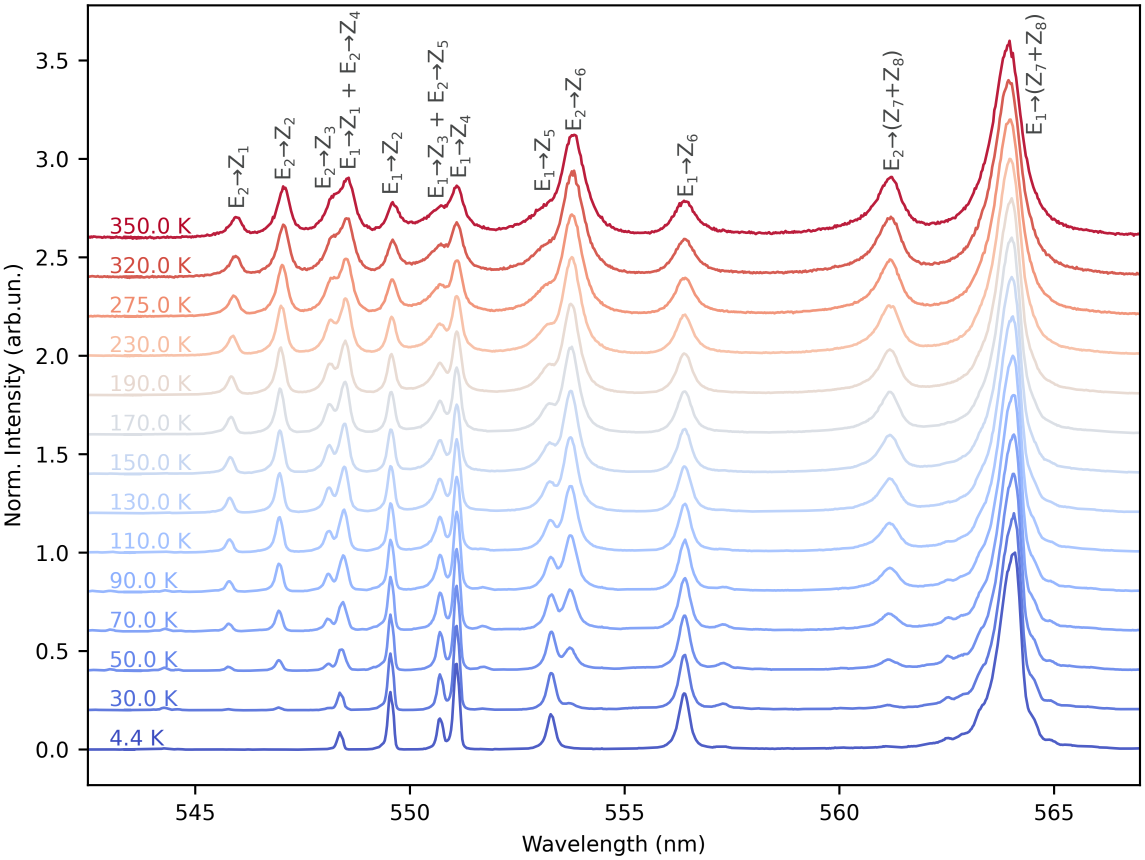

Figure S1: Normalized spectra of the $^4S_{3/2} \rightarrow {}^4I_{15/2}$ luminescence band for various temperatures between 4.4 K and 350.0 K (offset for clarity). The assignments follow the empirical notation as detailed in the main text.

Note that some of the lines - in addition to temperature-induced broadening - experience minor spectral shifts of up to 5 cm^{-1} when going from cryogenic to ambient temperatures, as also reported in other works.[S1] Furthermore, certain transitions originating from E_1 (e.g., $E_1 \rightarrow Z_6$) do not follow the same temperature dependence as the brightest peak, likely caused by a temperature

dependence of the radiative decay rate. We attribute both of these changes to the lattice expansion of the surrounding yttria, since the crystal fields impact the resulting Stark wavefunctions.[S2]

S2 Integrated intensity

Conventional ratiometric luminescence thermometry relies on the integrated intensities of the two manifold transitions $^2H_{11/2} \rightarrow {}^4I_{15/2}$ and $^4S_{3/2} \rightarrow {}^4I_{15/2}$. Fig. S2 shows their temperature-dependent count rates. As expected, the $^2H_{11/2}$ manifold has a negligible contribution to the emission spectra below 200 K, limiting its usefulness in cryogenic thermometry.

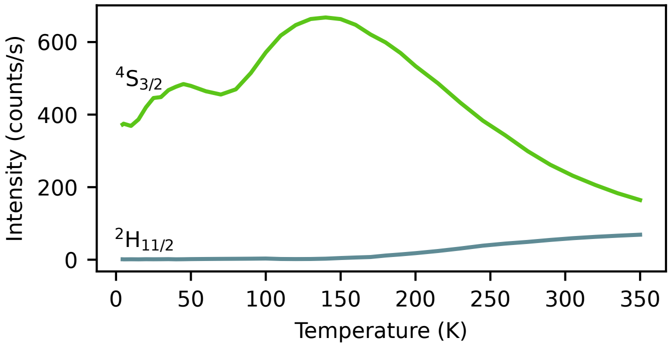

Figure S2: Integrated intensities of the $^2H_{11/2} \rightarrow {}^4I_{15/2}$ and $^4S_{3/2} \rightarrow {}^4I_{15/2}$ luminescence bands. For each temperature, the spectrum was integrated over 515 nm − 542 nm for the former and 542 nm − 580 nm for the latter.

S3 Stark lines deconvolution process

To accurately extract intensity ratios from partially overlapping Stark lines, simple spectral integration is insufficient due to finite linewidths and overlap from neighboring transitions. These linewidths arise from both homogeneous and inhomogeneous broadening: the former leads to Lorentzian line shapes, while the latter yields Gaussian components. Accordingly, we fit the emission lines with Voigt profiles, defined as the convolution:

$$V(x) = V_0 \int_{-\infty}^{\infty} G\left(x'; \sigma\right) L\left(x - x'; \gamma\right) \mathrm{d}x' \,, \tag{S1}$$

where $G(x;\sigma) = \frac{1}{\sigma\sqrt{2\pi}} \exp\left(-\frac{(x-x_0)^2}{2\sigma^2}\right)$ is the Gaussian component and $L(x;\gamma) = \frac{\gamma/\pi}{(x-x_0)^2+\gamma^2}$ is the

Lorentzian. V_0, x_0, σ and γ are the fitting parameters.

Fig. S3 shows the fluorescence spectrum at 100 K with overlaid Voigt fits for each resolvable transition; the two unfitted peaks (marked with an asterisk) contain overlapping contributions from at least three levels, and were therefore excluded from the discussions in the main manuscript due to a higher uncertainty in their fit. It is worth noticing that background correction prior to spectral deconvolution is essential to avoid contamination in the spectrum, which greatly impacts the temperature readout. We measured the background counts before the set of spectral acquisitions (with the same integration time) and removed it from the spectra afterward. Any reminiscent background due to CCD dark counts was removed by subtracting a linear baseline. For each temperature point, we measured the spectrum twice to remove any possible spurious cosmic rays.

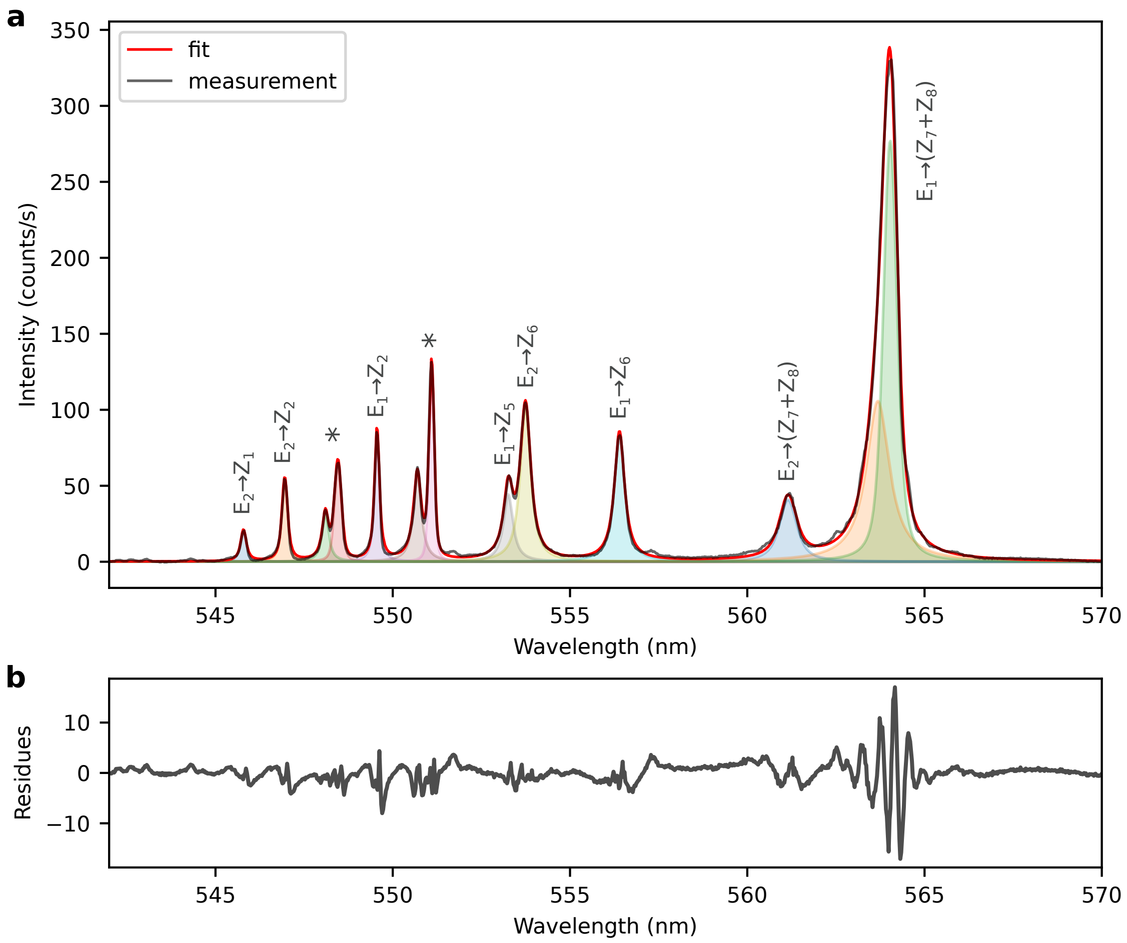

Figure S3: **a**, Emission spectrum of the $^4S_{3/2} \rightarrow {}^4I_{15/2}$ band at 100 K, with overlaid fitted Voigt profiles. The red line denotes the sum of the fitted profiles. Peaks used in the analysis are labeled; the ones marked with an asterisk (*) are excluded. **b**, Residues of the fit at 100 K.

S4 Power law

To avoid disruption of Boltzmann thermalization due to excited-state absorption, the excitation power has to be lower than the saturation threshold. We measured the overall intensity (I) of the $^2H_{11/2} \rightarrow {}^4I_{15/2}$ and $^4S_{3/2} \rightarrow {}^4I_{15/2}$ luminescence bands for a set of excitation irradiances (P) and obtained $I \propto P^n$, with $n = 2$, as shown in Fig S4**a**, for both transitions at room temperature. Fig. S4**a** also show the power law for the transition $^4S_{3/2} \rightarrow {}^4I_{15/2}$ at 4.4 K, where $n = 1.83$.

Also, to verify that self-heating effects are negligible – as it would influence the C_{eff} and ΔE_{eff} parameters – Fig. S4**b** shows R_{manifold} as a function of the excitation irradiance. The dashed line represents a linear fitting ($R_{\text{manifold}} = a \cdot P + b$), where we obtained only a minor power dependence of $a = 8 \times 10^{-4}$ (kW/cm^2)$^{-1}$ and $b = 0.2447$.

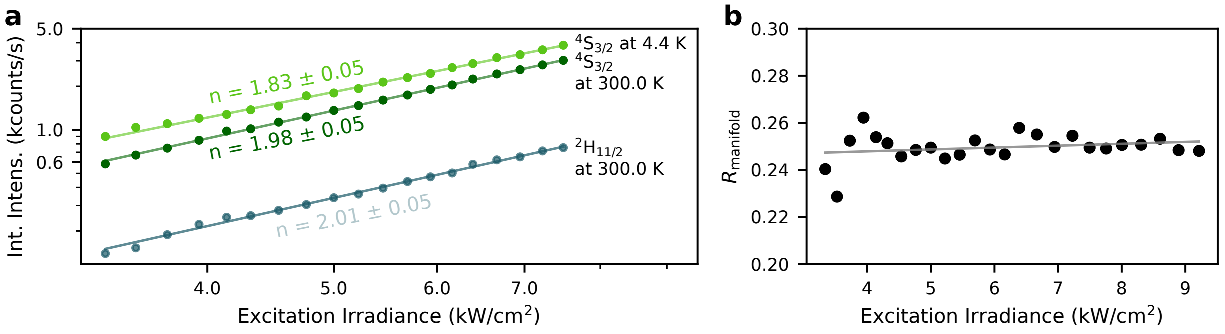

Figure S4: **a**: Integrated intensities of the luminescence bands as a function of the excitation irradiance (dots). Dashed lines represent a power-law fitting $I = I_0 P^n$. The resulting n fitted is shown within the plot. **b**: R_{manifold} as a function of the excitation irradiance, measured at 300 K. The dashed line represents a linear fitting $R_{\text{manifold}} = aP + b$.

S5 Error analysis

Since the integral $\int_{-\infty}^{\infty} V(x)\mathrm{d}x$ over a Voigt profile $V(x)$, as defined in section S3, yields its parameter V_0 (eq. S1), we considered the uncertainty of the measured Stark lines' spectrally integrated intensities as the uncertainty of the fitted parameter V_0, taken from the fitting algorithm. It already takes into account the residues of the experimental curve. Note that the correlation between the fitting parameters (V_0, x_0, σ and γ) must be properly handled. The lower V_0, the higher its relative uncertainty due to the shot noise limit.

The experimental R_{Stark} (Eq. 6 of the main manuscript) was computed as the ratio between

the spectrally integrated intensities (V_0) of the corresponding Stark-Stark lines. Its uncertainty is calculated based on conventional error propagation analysis. Similarly for ΔE_{eff} and C_{eff}, which come from the $\ln R_{\text{Stark}}$ *vs.* T^{-1} fitting. The cryostat temperature uncertainty is negligible in our case since its accuracy is in the order of mK, as mentioned in the main manuscript.

S6 Sample morphology and composition

The synthesized yttria nanoparticles doped with Yb^{3+} / Er^{3+} were characterized via X-ray diffraction (XRD). Measurements were conducted on a Siemens Bruker D5005 diffractometer operating with CuKα radiation ($\lambda = 1.5418\,\text{Å}$) and equipped with a graphite monochromator. The diffractogram was recorded with a step of $0.02\,^\circ\,\text{s}^{-1}$ in the 2θ range of $10\,^\circ\,\text{s}^{-1}$ to $90\,^\circ\,\text{s}^{-1}$ (Fig. S5a). The pattern shows good agreement with the reference spectrum for undoped cubic-phase yttria, confirming that the synthesized material retains a predominantly cubic crystalline structure.

Their morphology was examined under a JEOL JEM-100CX II Transmission Electron Microscope (TEM) operating at 100 kV (Fig. S5b). Ethanol was used to disperse the particles, allowing to measure their sizes. Average extracted diameters were $80 \pm 10\,\text{nm}$.

Changes in morphology and size may slightly alter the Stark sublevel energies, but not compromise the characteristic emission spectrum of the Yb^{3+} and Er^{3+} dopants in Y_2O_3 with similar results from 10 nm particles[S3] to crystals with hundreds of nm in diameter,[S4] or distinct morphologies[S5] and even spherical microspheres.[S6] The size may imply some changes in the population mechanisms due to quenching effects related to surface defects. However, these size-dependent effects are only relevant in small particles of diameters below 20 nm,[S7,S8] which is not the case presented in our work.

Similarly, the dopant concentrations of Er^{3+} and Yb^{3+} (0.5 mol% and 1.5 mol%, respectively, relative to the molar concentration of Y^{3+}), do not impact thermometric characteristics as long as they remain at low levels, as has been shown for Er^{3+} ions[S9] and Yb^{3+} in similar crystals.[S10] Only at concentrations above 2 % for Er^{3+} ions and 10 % for Yb^{3+} ions, excited state absorption and cross-relaxation processes start impacting the performance of luminescent nanothermometers.[S11,S12]

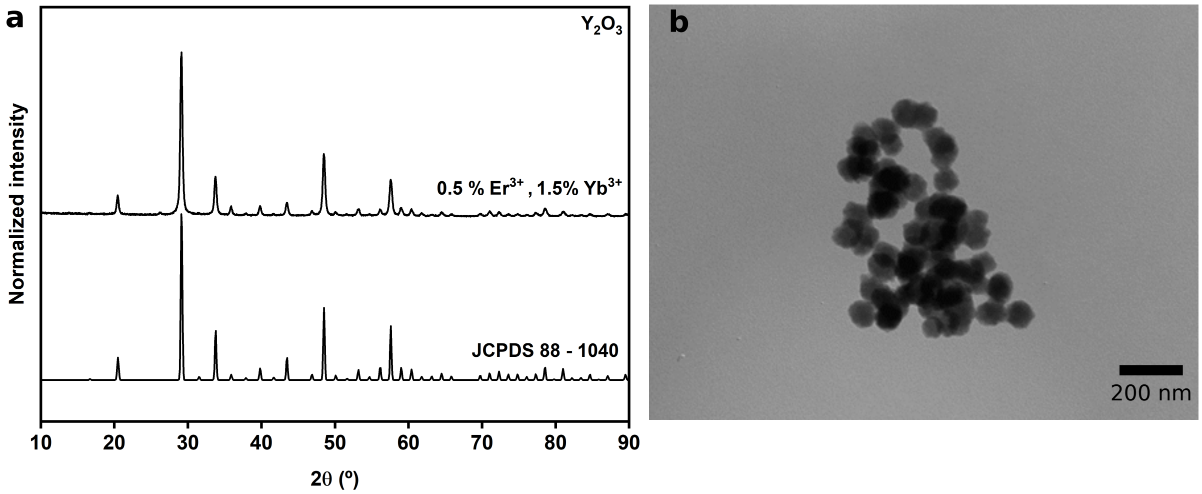

Figure S5: **a**, X-ray diffractogram of the Y_2O_3: Yb^{3+} / Er^{3+} nanoparticles used in the experiments, with the corresponding reference spectrum for pure Y_2O_3 in its cubic phase (JCPDS 88-1040). **b**, TEM image showing particle morphology.

S7 Experimental setup

To characterize the sample at cryogenic temperatures, it was mounted in a liquid helium cryostat. Illumination was performed in reflection geometry (Fig. S6), using a tunable Ti:Sapphire laser emitting 140 fs pulses at a central wavelength of 980 nm.

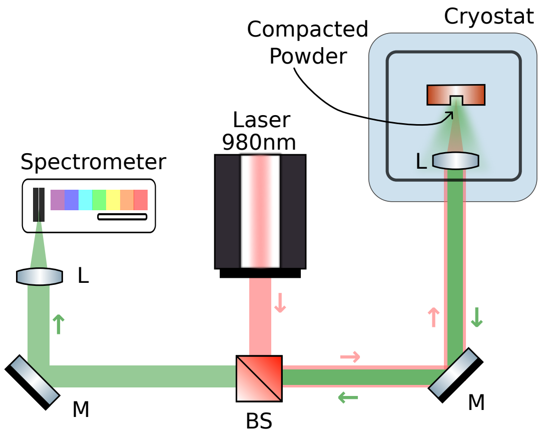

Figure S6: Schematic of the experimental setup for thermometric measurements. L: lens, M: mirror, BS: beam splitter.

References

(S1) Dodson, A.; Wu, H.; Rai, A.; Apte, S.; O'Hara, A.; Lawrie, B.; Wang, Y.; Ueda, A.; Krzyżanowska, H.; Titze, M. *et al.* Phonon-mediated temperature dependence of Er^{3+} optical transitions in Er_2O_3. *Communications Physics* **2024**, *7*.

(S2) Ćirić, A.; Stojadinović, S.; Dramićanin, M. D. Temperature and concentration dependent Judd-Ofelt analysis of Y_2O_3:Eu^{3+} and YVO_4:Eu^{3+}. *Physica B: Condensed Matter* **2020**, *579*, 411891.

(S3) Bai, X.; Song, H.; Pan, G.; Lei, Y.; Wang, T.; Ren, X.; Lu, S.; Dong, B.; Dai, Q.; Fan, L. Size-Dependent Upconversion Luminescence in Er^{3+}/Yb^{3+}-Codoped Nanocrystalline Yttria: Saturation and Thermal Effects. *The Journal of Physical Chemistry C* **2007**, *111*, 13611–13617.

(S4) Lojpur, V.; Mancic, L.; Vulic, P.; Dramicanin, M.; Rabanal, M.; Milosevic, O. Structural, morphological and up-converting luminescence characteristics of nanocrystalline Y_2O_3:Yb/Er powders obtained via spray pyrolysis. *Ceramics International* **2014**, *40*, 3089–3095.

(S5) Lojpur, V.; Ahrenkiel, S. P.; Dramićanin, M. D. Yb^{3+}, Er^{3+} doped Y_2O_3 nanoparticles of different shapes prepared by self-propagating room temperature reaction method. *Ceramics International* **2014**, *40*, 16033–16039.

(S6) Guo, H.; Qiao, Y. M. Preparation, characterization, and strong upconversion of monodisperse Y_2O_3:Er^{3+}, Yb^{3+} microspheres. *Optical Materials* **2009**, *31*, 583–589.

(S7) Zhao, J.; Lu, Z.; Yin, Y.; McRae, C.; Piper, J. A.; Dawes, J. M.; Jin, D.; Goldys, E. M. Upconversion luminescence with tunable lifetime in NaYF4:Yb, Er nanocrystals: role of nanocrystal size. *Nanoscale* **2013**, *5*, 944–952.

(S8) Marciniak, L.; Bednarkiewicz, A.; Strek, W. The impact of nanocrystals size on luminescent properties and thermometry capabilities of Cr, Nd doped nanophosphors. *Sensors and Actuators B: Chemical* **2017**, *238*, 381–386.

(S9) Zhou, J.; Chen, Y.; Lei, R.; Wang, H.; Xu, S. Role of Er^{3+} concentration on the photoluminescence and temperature sensing properties in Er^{3+} doped Y_2O_3 based transparent ceramics. *Journal of Luminescence* **2019**, *215*, 116671.

(S10) He, Y.; Lei, R.; Zhao, S.; Xu, S. Effects of Yb^{3+} concentration and excitation power on thermally enhanced upconversion luminescence and optical thermometric properties in $Sc_2Mo_3O_{12}$: Er^{3+}/ Yb^{3+}. *Journal of Luminescence* **2025**, *281*, 121137.

(S11) Baride, A.; May, P. S.; Berry, M. T. Cross-Relaxation from $Er^{3+}(^2H_{11/2}, {}^4S_{3/2})$ and $Er^{3+}(^2H_{9/2})$ in β-$NaYF_4$:Yb, Er and Implications for Modeling Upconversion Dynamics. *The Journal of Physical Chemistry C* **2019**, *124*, 2193–2201.

(S12) Kumar, V.; Bullis, G.; Wang, G. Investigation of NIR-to-red upconversion luminescence mechanism in Y_2O_3:Er^{3+}, Yb^{3+} and the effect of co-doping Zn in the matrix. *Journal of Luminescence* **2017**, *192*, 982–989.
